# *Pseudomonas fluorescens* and *Escherichia coli* in Fresh Mozzarella Cheese: Effect of Cellobiose Oxidase on Microbiological Stability during Refrigerated Shelf Life

**DOI:** 10.3390/foods12010145

**Published:** 2022-12-27

**Authors:** Martina Marrella, Gaia Bertani, Annalisa Ricci, Rossana Volpe, Sebastien Roustel, Federico Ferriani, Elia Nipoti, Erasmo Neviani, Camilla Lazzi, Valentina Bernini

**Affiliations:** 1 Department of Food and Drug, University of Parma, 43124 Parma, Italy; 2 Chr. Hansen A/S, 2970 Hørsholm, Denmark; 3 Chr. Hansen Italia, 43126 Parma, Italy; 4 Interdepartmental Center, SITEIA.PARMA—Centro Interdipartimentale sulla Sicurezza, Tecnologie e Innovazione Agroalimentare, University of Parma, 43124 Parma, Italy

**Keywords:** cellobiose oxidase, mozzarella cheese, antimicrobial activity, *Escherichia coli*, *Pseudomonas fluorescens*

## Abstract

Background: Mozzarella cheese possesses a high moisture content (50–60%) and a relatively high pH (around 5.5) and is therefore considered a perishable food product characterized by high quality deterioration and the potential risk of microbial contamination. Moreover, it can be spoiled by *Pseudomonas* spp. and coliform bacteria, which may be involved in different negative phenomena, such as proteolysis, discolorations, pigmentation, and off-flavors. To prevent these, different methods were investigated. In this context, the present study aims to assess the antimicrobial effect of cellobiose oxidase on *Pseudomonas fluorescens* (5026) and *Escherichia coli* (k88, k99) in mozzarella cheese during refrigerated shelf life. Methods: microbiological challenge tests were designed by contaminating the mozzarella covering liquid containing different cellobiose oxidase concentrations with *P. fluorescens* (5026) and *E. coli* (k88, k99). The behavior of these microorganisms and the variation of hydrogen peroxide concentrations were then tested under refrigerated conditions for 20 days to simulate the mozzarella cheese shelf life. Results and Conclusions: The data obtained demonstrated the effect of cellobiose oxidase on microbial growth. In particular, *E. coli* (k88, k99) was inhibited over the entire shelf life, while *P. fluorescens* (5026) was only partially affected after a few days of refrigerated storage.

## 1. Introduction

Mozzarella is a soft, unripened “pasta filata” cheese with an ancient origin that is traditionally produced in southern Italy [1]. Nevertheless, this product is widespread all over the world and extremely appreciated for its soft full-bodied fragrance and its fibrous structure. Mozzarella cheesemaking provides the use of pasteurized bovine or buffalo milk and the enforcement of two different acidification steps before rennet addition [2]. In direct acidification, food-grade organic acids (usually citric acid and less frequently lactic acid) are added, leading to a quick-stretch curd after coagulation. On the other hand, mozzarella manufacturing can also provide the use of defined cultures cultivated in milk or direct inoculation (DVS) resulting from the thermophilic microorganisms’ growth, which gives rise to microbial acidification [3]. When the cheesemaking process ends, the mozzarella cheese is submerged in covering liquid and stored in refrigerated condition [4]. Over the years, several studies have described the microbiota present in this matrix [5,6,7]. Mozzarella cheese is a complex ecosystem, characterized by a heterogeneous microbial consortium, composed by several species such as *Lactococcus lactis* subsp. *lactis*, *L. lactis* subsp. *diacetylactis*, *L. lactis* subsp. *cremoris*, *Streptococcus thermophilus*, *Enterococcus faecium*, *E. faecalis*, *Escherichia coli*, some yeasts, and various spoilage psychrophilic microbiota [8,9,10,11]. Due to this microbial complexity and the high moisture content (50–60%) associated with a relatively high pH (around 5.5), mozzarella cheese is characterized by a high quality deterioration and by the potential risk of microbial contamination [12]. Several studies highlighted that this product can be spoiled by *Pseudomonas* spp. and coliform bacteria, which may be involved in different negative phenomena, such as proteolysis, discolorations, pigmentation, and off-flavors [6,9,13,14,15]. *Pseudomonas* spp., probably derived from the water used during the cheesemaking process, can grow on the cheese surface. On the other hand, *E. coli,* belonging to coliforms, is widely found as a commensal microorganism in the human and animal gut, and it is considered an indicator of microbiological quality and hygiene in food products [16]. In recent years, several studies aimed to extend mozzarella cheese shelf life, since it normally ranges from approximately 5 to 10 days depending on the microbial content, moisture content, manufacturing procedures, and storage conditions [17]. It has been demonstrated that different treatments, such as carbon-dioxide-based modified atmospheres [18], lysozyme combined with Na-EDTA [19], natural antimicrobial compounds [20], and chitosan [12], can carry out an important role against molds, yeasts, spoilage, Gram-positive bacteria, coliforms, and *Pseudomonas* spp. Additionally, the use of milk proteins, which turned out to have antimicrobial activity against Gram-positive and Gram-negative pathogens, fungi, and viruses, were proposed as preservatives in foodstuffs [21,22,23]. The use of these substances and the innovation of procedures involved in mozzarella production, such as the replacement of covering liquid with a polysaccharide-based gel, resulted in useful strategies to extend the shelf life of the product [24,25]. Recently, following this direction, the antimicrobial and antioxidant activity of cellobiose oxidase has been proven (CDH) (EC1.1.99.18) [26]. Cellobiose oxidase is a dehydrogenase isolated from *Basidiomycota* and *Ascomycota* fungi [27,28] that catalyzes the oxidation of cellobiose, di- or oligosaccharides characterized by β-1,4-glycosidic bonds and sometimes also monosaccharides. The products obtained from this reaction have a different fate. The aldono-1,5- lactones are hydrolyzed spontaneously to aldonic acids in aqueous solutions, while the cofactor FADH_2_ is reoxidized by one or two-electron acceptors [29,30,31,32]. Lastly, the electrons produced react with molecular oxygen, leading to the production of hydrogen peroxide (H_2_O_2_) [33]. Recently, available reports have highlighted that CDH plays a key role in various fields such as biotechnology, pharmaceuticals, clinical fields, cosmetics, and food [34]. In addition, the antimicrobial role of H_2_O_2_ is well-known and can modify the structure and the permeability of the cell wall, protein synthesis, and energy production and can cause DNA damage [34,35,36]. Given the recently discovered effect on CDH antimicrobial activity, this paper, for the first time, to the best of our knowledge, aims to assess the antimicrobial effect of CDH on *Pseudomonas fluorescens* (5026) and *Escherichia coli* (k88, k99) in mozzarella cheese during its refrigerated shelf life. To reach this purpose, microbiological challenge tests (MCT) were designed by contaminating mozzarella covering liquid containing different CDH concentrations with *P. fluorescens* (5026) and *E. coli* (k88, k99) separately. The behavior of these microorganisms and variations in hydrogen peroxide concentrations were then tested under refrigerated conditions for 20 days to simulate the mozzarella cheese shelf-life. 

## 2. Materials and Methods

### 2.1. Samples

A whole amount of 92 multipacks, each containing 5 mozzarellas (each one weighing 50–60 g) submerged in 250–300 mL of covering liquid, were manufactured and provided by San Martino srl (Trana, Turin, Italy). Samples were kept in refrigerated conditions (4.0 ± 1.8 °C) until use. 

### 2.2. P. fluorescens and E. coli Strains

One strain of *P. fluorescens* (5026) and a co-culture composed of two non-pathogenic *E. coli* strains (k88, k99), all stored in the University of Parma Culture Collection (UPCC), were used as target strains for MCT design. Before use, each strain, kept at −80 °C in Tryptone Soya Broth (TSB) (Oxoid, Basingstoke, UK) supplemented with 12.5% glycerol (*v/v*), was revitalized twice by 3% (*v/v*) inoculum in TSB (Oxoid) and then incubated for 16 h at 25 °C and 37 °C, respectively, for *P. fluorescens* and *E. coli* in aerobic conditions.

### 2.3. Microbiological Challenge Tests

In total, 40 multipacks were contaminated with *P. fluorescens* (5026) and 40 multipacks with *E. coli* (k88, k99) conversely, the last 12 multipacks were used as blank samples. Different experimental designs were planned to investigate the antimicrobial activity of CDH toward *P. fluorescens* and *E. coli*. Briefly, the covering liquid of each multipack was contaminated separately with *P. fluorescens* and with the co-culture of *E. coli* strains employed in the same concentrations to obtain for each target species two final concentrations of 2 Log CFU/mL (L1) and 4 Log CFU/mL (L2). Moreover, different concentrations of CDH (LactoYIELD^®^, Chr. Hansen, Denmark) (A: 100 LOX-U/100 L, B: 150 LOX-U/100 L and C: 200 LOX-U/100 L) were added to the contaminated samples. Blank samples (not contaminated and not containing CDH) and control samples (contaminated but not containing CDH) were included in the experimental design. Each multipack was stored by simulating the shelf life of mozzarella cheese in refrigerated conditions (4.0 ± 1.8 °C). The five mozzarellas contained in all the contaminated multipacks were analyzed just after artificial inoculum (t0) and after 5 (t5), 8 (t8), 10 (t10), 15 (t15), and 20 (t20) days of storage. Blank and control samples were analyzed five times at the same times. 

### 2.4. Microbiological Analysis

The concentrations of *P. fluorescens* and *E. coli* were examined in mozzarella by plate count at established times reported in Section 2.3. Samples were homogenized 1:10 (v:v) in Ringer solution (VWR, Radnor, PA, USA) then 10-fold serially diluted in Ringer (VWR), and dilutions were inoculated on appropriated agar media. *P. fluorescens* concentration was quantified using Pseudomonas agar base (Oxoid Ltd., Basingstoke, UK) added with CFC supplement (Oxoid), as reported by da Silva Rodrigues et al., 2021 [37], and characteristic colonies were checked after incubation at 25 °C for 48 h. *E. coli* concentrations were assessed on Chromocult Agar medium (Merck, Darmstadt, Germany) after plate incubation at 37 °C for 48 h [38]. 

Simultaneously, in blank samples not artificially contaminated, the total mesophilic microbiota naturally present in mozzarella were detected on Plate Count Agar medium (PCA) (VWR, Radnor, PA, USA) after incubation of the plates at 30 °C for 48 h [39]. The concentrations of *Pseudomonas* spp. and coliform bacteria were also determined by applying the same method employed for the detection of *P. fluorescens* and *E. coli*. 

The data obtained were reported as the mean value ± standard deviation and the differences were statistically evaluated through one-way ANOVA test, and mean separation was performed with Tukey’s test (*p* ≤ 0.05). The differences in microbial concentration observed over time were analyzed within the samples inoculated with the same initial concentration. All the analyses were carried out using SPSS Statistics 23.0 software (SPSS Inc., Chicago, IL, USA).

### 2.5. Determination of Hydrogen Peroxide Concentration

The concentration of H_2_O_2_ was detected in triplicate in samples of mozzarella for all the conditions proposed in the microbiological challenge test at the time scheduled in Section 2.3. Peroxide Assay kit (Sigma-Aldrich) was used for this purpose according to the protocol proposed by the producer, and H_2_O_2_ concentration was reported as μM using a standard curve (H_2_O_2_) for quantification.

## 3. Results

### 3.1. Evaluation of Endogenous Microbiota of Mozzarella Cheese

The concentrations of total mesophilic microbiota, *Pseudomonas* spp., and coliform bacteria were evaluated in blank samples to follow up the behavior of endogenous contamination of mozzarella during the refrigerated shelf life (0, 5, 8, 10, 15, 20 days). Regarding the total mesophilic microbiota, the initial concentration was 4 Log CFU/g, reaching 8 Log CFU/g after 8 days of storage and then remaining stable until the end of the shelf life. Instead, *Pseudomonas* spp. was initially present at 1.6 Log CFU/g, while it was detected at about 7 Log CFU/g after 20 days of refrigerated storage. Similar to the concentration of *Pseudomonas* spp., the concentration of endogenous coliform bacteria was lower than 1 Log CFU/g at the beginning of the storage, increasing to reach the concentration of about 4 Log CFU/g after 20 days.

### 3.2. Effect of CDH toward P. fluorescens and E. coli in Mozzarella Cheese

To evaluate the antimicrobial effect of a CDH standardized solution toward *P. fluorescens* and *E. coli*, the target species inoculated into the covering liquid were followed up at 5, 8, 10, 15, and 20 days of refrigerated storage of mozzarella. In particular, the effect of three CDH concentrations and two different artificial contamination levels for each target (about 2 Log and 4 Log CFU/mL) in the covering liquid were considered. 

#### 3.2.1. Behavior of *P. fluorescens* in Mozzarella Cheese during Refrigerated Shelf Life

All the samples analyzed that were inoculated with *P. fluorescens* (added or not with CDH) revealed that this microorganism is able to grow in mozzarella cheese in refrigerated conditions. In detail, *P. fluorescens* concentration increased in control samples (not added with CDH) over time, reaching a concentration higher than 7 Log CFU/g after 8 days and then remaining almost stable until the end of shelf life. Instead, in artificially inoculated samples containing different CDH concentrations, a slight decrease in *P. fluoescens* concentration was observed. A slight, but in some cases not significant, antimicrobial effect of the enzyme was noticeable after 5 days and compared to samples not added with CDH at the end of storage. The results observed highlighted that the same behavior was observed for all the contamination levels, with no particular differences between the different concentrations tested (Table 1). 

#### 3.2.2. Behavior of *E. coli* in Mozzarella Cheese during Refrigerated Shelf Life

Similar to *P. fluorescens*, *E. coli* was able to grow in refrigerated conditions in the absence of CDH. Its concentration increased over time, reaching about 6 Log CFU/mL after 5 days, decreasing to about 0.5 Log CFU/mL after 15 days, and then remaining stable until the end of storage. Differently from *P. fluorescens*, the antimicrobial activity of CDH was evident against *E. coli*. Indeed, for both artificial contamination levels tested (2 Log and 4 Log CFU/mL), the initial concentration decreased towards the detection limit at the end of the shelf life. This decrease was not highlighted in control samples (L1, L2), where, at the end of shelf life, *E. coli* reached a concentration of about 5 Log CFU/g. The bactericidal effect of the lowest CDH concentration (100 LOX-U/100L) was evident at T20 in the presence of the lowest inoculum level (L1A) and at T10 with higher enzyme concentrations (150 LOX-U/100L, L1B; 200 LOX-U/100L, L1C). In detail, for L1A, L1B and L1C, a downward trend can be observed during the various samplings, reaching, at the end of storage, values below 1 Log CFU/g for all the CDH concentrations tested. On the other hand, with a higher *E. coli* contamination (L2), the antimicrobial effect of CDH was particularly evident in the presence of the highest CDH concentrations (200 LOX-U/100L, L2C) (Table 2).

**Table 1:** The behavior of *P. fluorescens* (5026) in mozzarella cheese (L1: 2 Log CFU/mL and L2: 4 Log CFU/mL) in presence of CDH (A: 100 LOX-U/100L, B: 150 LOX-U/100L, C: 200 LOX-U/100L) during refrigerated shelf life (data are reported as the mean values ± standard deviation of Log CFU/g). The differences in microbial concentration observed over time were analyzed within the samples inoculated with the same initial concentration. Means with the same letter are not significantly different.

	L1			L1 A			L1 B			L1 C			L2			L2 A			L2 B			L2 C		
T0	1.92	±	0.10 ^a^		/			/			/		3.81	±	0.14 ^h^		/			/			/	
T5	>4			3.43	±	0.66 ^b^	3.34	±	0.56 ^b^	4.31	±	0.66 ^b^	> 6			4.53	±	0.56 ^i^	4.47	±	0.48 ^i^	4.30	±	0.97 ^i^
T8	7.43	±	0.15 ^cfg^	6.10	±	0.29 ^cd^	6.04	±	0.58 ^cd^	5.46	±	0.42	7.44	±	0.11 ^pq^	5.12	±	0.36 ^lmn^	6.73	±	0.33 ^mnop^	6.69	±	0.02 ^lm^
T10	7.48	±	0.06 ^cfg^	5.45	±	0.63	6.61	±	0.22 ^de^	6.29	±	0.32 ^de^	7.58	±	0.11 ^q^	6.37	±	0.40 ^lmnoq^	6.62	±	0.03 ^nopq^	6.47	±	0.35 ^mnopq^
T15	7.43	±	0.12 ^fg^	6.83	±	0.17 ^def^	6.91	±	0.32 ^ef^	6.90	±	0.09 ^ef^	7.18	±	0.11 ^pq^	7.40	±	0.08 ^p^	7.04	±	0.15 ^opq^	7.05	±	0.15 ^op^
T20	7.76	±	0.09 ^g^	6.81	±	0.23 ^def^	6.88	±	0.10 ^ef^	6.87	±	0.26 ^ef^	7.38	±	0.14 ^q^	6.91	±	0.19 ^opq^	7.00	±	0.09 ^opq^	6.64	±	0.13 ^opq^

### 3.3. Hydrogen Peroxide Concentrations in Mozzarella 

**Figure 1:**
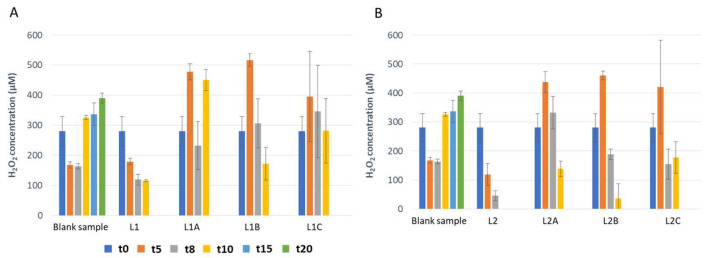
Hydrogen peroxide concentration examined in mozzarella samples contaminated with *P. fluorescens* (5026) over the shelf life. The covering liquid was inoculated with 2 Log CFU/mL (**A**) and 4 Log CFU/mL (**B**).

**Figure 2:**
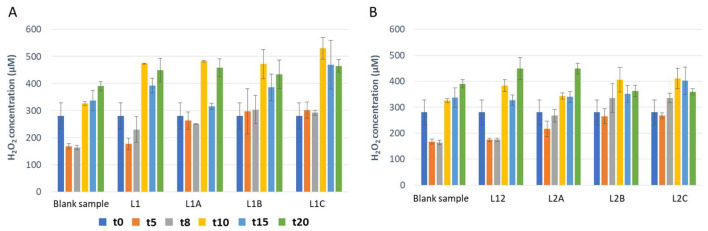
Hydrogen peroxide concentration examined in mozzarella samples contaminated with *E. coli* (k88, k99) over the shelf life. The covering liquid was inoculated with 2 Log CFU/mL (**A**) and 4 Log CFU/mL (**B**).

In addition to following up the behavior of target species microorganisms in mozzarella samples, hydrogen peroxide concentration was also tested in mozzarella samples during the refrigerated shelf life. Overall, in the samples contaminated with *P. fluorescens* a decrease in hydrogen peroxide concentrations was observed during the shelf life, especially after 10, 15 and 20 days (Figure 1). This decrease seems to be related to the increase in the high concentration of *P. fluorescens*. Indeed, a high concentration of *P. fluorescens* corresponds to a low concentration of hydrogen peroxide (Table 1 and Figure 1). Otherwise, in those samples contaminated with *E. coli*, the opposite trend can be observed, and the increase in hydrogen peroxide concentration is noticeable over the shelf life (Figure 2). Indeed, as can be deduced from Table 2 and Figure 2, in samples containing *E. coli* the increase of hydrogen peroxide seems to be linked with the inhibition of the same microorganism.

## 4. Discussion

Among food products, mozzarella cheese is recognized to be a perishable foodstuff and can be contaminated by spoilage or pathogenic microorganisms. The prevalent cause of deterioration is the increase in microorganisms present in the product which can be responsible for the additional acidification, the development of off-flavors, and the coloration of the mozzarella pasta [40]. In particular, in mozzarella cheese, the addition of preservatives in covering liquid was exploited by different authors [17]. Different antimicrobial compounds, such as NaCl, CaCl, MgCl, etc., were employed to extend the shelf life and to ensure a safe product [4,40]. In order to assess the microbial quality and safety of dairy products, indicator organisms must be evaluated. Coliform bacteria, belonging to the Enterobacteriaceae family, are considered indicator organisms for fecal and environmental contamination, while Pseudomonas spp. may be evaluated as an important indicator to follow up the plant environment [41]. Following these considerations, in this paper the attention was focused on the enzyme cellobiose oxidase (CDH) that was added to the covering liquid of mozzarella cheese and whose antimicrobial activity was evaluated to determine its efficacy against *E. coli* (k88, k99) and *P. fluorescens* (5026) until 20 days of refrigerated storage. CDH is a dehydrogenase that catalyzes the oxidation of cellobiose, di- or oligosaccharides, and sometimes also monosaccharides. During sugar oxidation, the electrons yielded can be transferred to molecular oxygen, reducing it in hydrogen peroxide [34]. So, the ability of CDH to reduce molecular oxygen to hydrogen peroxide can be employed to block the growth of undesirable microorganisms in food products. Hydrogen peroxide is a well-known antimicrobial agent since it is a strong oxidative agent. The antimicrobial activity of hydrogen peroxide is related to the peroxidation and disruption of membrane layers, enzyme inhibition, and oxidation of oxygen scavengers and thiol groups; moreover, it can also oxidate the nucleosides and the protein synthesis can be blocked leading to the death of the cell [34,35]. Moving from these premises, in the present article, the antimicrobial activity of CDH was tested against *P. fluorescens*, noted for its spoilage activity, and *E. coli*, a microorganism used as an indicator for fecal contamination. *P. fluorescens* (5026) was able to grow and proliferate also in the presence of CDH at different concentrations (100 LOX-U/100L, 150 LOX-U/100L, 200 LOX-U/100L), even if a slight decrease can be observed comparing its concentration with the concentrations of control samples (samples added with *P. fluorescens* (5026) but non containing CDH). Instead, *E. coli* (k88, k99) growth was more affected by CDH, reducing it by about 4–5 Log CFU/mL depending on the initial concentration. Different authors have connected the antimicrobial activity of CDH with the production of hydrogen peroxide [34,42], so it can be speculated that the antimicrobial activity observed is connected to the presence of this compound. However, despite both *P. fluorescens* and *E. coli* being catalase producer species [43,44], their growth was differently affected by CDH. Theoretically, catalase producers’ microorganisms are able to degrade the hydrogen peroxide by converting it into oxygen and water [34], blocking the antimicrobial activity of hydrogen peroxide. Indeed, *P. fluorescens* was only slightly affected by the presence of CDH. Different strains of the same genus showed similar results. *Pseudomonas aeruginosa* was only moderately inhibited by high concentrations of CDH (in culture medium) [42], while another study demonstrates that the expression of different genes involved in catalase production is essential for the survival of *P. aerugiosa* in presence of hydrogen peroxide [45]. Conversely to *P. fluorescens* (5026), *E. coli* (k88, k99) showed to be more affectable by hydrogen peroxide. Nyanhongo et al. [34] suggested that the damage can be ascribed to the loss of outer structural integrity and the increase in cell permeability. The same authors reported that low levels of hydrogen peroxide induce the cells to form filaments and to release a limited quantity of intracellular components, while, at a high concentration, there is the loss of intracellular compounds with the reduction of cell volume. Some other studies proposed that the antimicrobial activity observed in honey can be related to the presence of hydrogen peroxide [46,47]. So, the presence of hydrogen peroxide or compounds that lead to the release of hydrogen peroxide in food matrices can exert a protective role against spoilage and pathogenic microorganisms even if at different levels, depending on the targeted species. 

## 5. Conclusions

Since the deterioration of food products has an economic impact on the producers, the study of new strategies for its prevention and the extension of shelf life is of increasing interest. To prevent the growth of spoilage microorganisms, several preservation techniques have been proposed such as the direct incorporation of the preservative into the food product formulation or into the packaging material. To the best of our knowledge, this is the first time that CDH was applied as a preservative in mozzarella cheese covering liquid. From the data obtained in the present work, CDH has demonstrated its ability to affect the growth, especially of *E. coli* (k88, k99) over the entire shelf life of 20 days. Otherwise, *P. fluorescens* (5026) was only partially inhibited after a few days of refrigerated storage. 

## Figures and Tables

**Table 2:** The behavior of *E. coli* (k88, k99) in mozzarella cheese (L1: 2 Log CFU/mL and L2: 4 Log CFU/mL) in presence of CDH (A: 100 LOX-U/100L, B: 150 LOX-U/100L, C: 200 LOX-U/100L) during refrigerated shelf life (data are reported as the mean values ± standard deviation of Log CFU/g). The differences in microbial concentration observed over time were analyzed within the samples inoculated with the same initial concentration. Means with the same letter are not significantly different.

	L1			L1 A			L1 B			L1 C			L2			L2 A			L2 B			L2 C		
T0	2.51	±	0.06 ^a^		/			/			/		3.65	±	0.17 ^fg^		/			/			/	
T5	6.03	±	0.31 ^b^	1.22	±	0.52 ^cde^	1.11	±	0.27 ^cde^	<1			6.59	±	0.18 ^n^	3.64	±	0.37 ^fghi^	3.45	±	0.21 ^fghil^	3.50	±	0.17 ^fghil^
T8	6.02	±	0.35 ^b^	1.99	±	0.56 ^ad^	1.95	±	0.92 ^cde^	1.05	±	0.14 ^de^	6.29	±	0.26 ^n^	4.41	±	0.47 ^fg^	3.45	±	0.31 ^fghil^	3.38	±	0.54 ^fghil^
T10	6.14	±	0.25 ^b^	2.44	±	0.68 ^a^	<1			<1			6.67	±	0.18 ^n^	4.79	±	0.15 ^f^	2.39	±	0.35 ^ghil^	2.76	±	0.61 ^ghil^
T15	5.45	±	0.31 ^b^	2.17	±	0.61 ^cd^	<1			<1			5.11	±	0.17 ^mn^	4.22	±	0.33 ^fghi^	2.05	±	0.14 ^hil^	1.91	±	0.46 ^il^
T20	5.63	±	0.33 ^b^	<1			<1			<1			5.08	±	0.34 ^mn^	3.17	±	0.26 ^fghil^	2.18	±	0.27 ^ghil^	1.07	±	0.12 ^l^

## Data Availability

Data is contained within the article.
